# 30 years of youth system of care lessons learned – a qualitative study of Hawaiʻi’s partnership with the Substance Abuse and Mental Health Services Administration

**DOI:** 10.1186/s12913-024-11114-9

**Published:** 2024-05-23

**Authors:** Kelsie H. Okamura, David Jackson, Danielle L. Carreira Ching, Da Eun Suh, Tia L. R. Hartsock, Puanani J. Hee, Scott K. Shimabukuro

**Affiliations:** 1 The Baker Center for Children and Families, Harvard Medical School, Cambridge, MA USA; 2 Department of Psychology, University of Hawaiʻi at Mānoa, Honolulu, HI USA; 3 Hawaiʻi State Office of Wellness and Resilience, Honolulu, HI USA; 4 Hawaiʻi State Child and Adolescent Mental Health Division, Honolulu, HI USA

**Keywords:** System of Care, Hawaiʻi, Substance Abuse and Mental Health Services Administration

## Abstract

**Background:**

The Hawaiʻi State Department of Health, Child and Adolescent Mental Health Division (CAMHD) has maintained a longstanding partnership with Substance Abuse and Mental Health Services Administration (SAMHSA) to enhance capacity and quality of community-based mental health services. The current study explored CAMHD’s history of SAMHSA system of care (SOC) awards and identified common themes, lessons learned, and recommendations for future funding.

**Methods:**

Employing a two-phase qualitative approach, the study first conducted content analysis on seven final project reports, identifying themes and lessons learned based on SOC values and principles. Subsequently, interviews were conducted with 11 system leaders in grant projects and SOC award projects within the state. All data from project reports and interview transcripts were independently coded and analyzed using rapid qualitative analysis techniques.

**Results:**

Content validation and interview coding unveiled two content themes, interagency collaboration and youth and family voice, as areas that required long-term and consistent efforts across multiple projects. In addition, two general process themes, connection and continuity, emerged as essential approaches to system improvement work. The first emphasizes the importance of fostering connections in family, community, and culture, as well as within workforce members and child-serving agencies. The second highlights the importance of nurturing continuity throughout the system, from interagency collaboration to individual treatment.

**Conclusions:**

The study provides deeper understanding of system of care evaluations, offering guidance to enhance and innovate youth mental health systems. The findings suggest that aligning state policies with federal guidelines and implementing longer funding mechanisms may alleviate administrative burdens.

**Supplementary Information:**

The online version contains supplementary material available at 10.1186/s12913-024-11114-9.

## Background

Youth are disproportionately impacted by mental health disorders with average rates higher than adults in the United States [1]. This begins early on with one in six children aged two to eight years diagnosed with a mental, behavioral, or developmental disorder and persists over time with one in five youth having experiences with a severe mental health disorder at some point in their life [1–3]. At the end of 2021, the U.S. Surgeon General declared a youth mental health crisis noting that rates of emergency room visits for suspected suicide attempts had increased in some demographics by more than 50% compared to the same time period in 2019 [4]. Despite the large and increasing need for services, alarming gaps have been found in access to care and it is estimated that half of youth will not receive adequate treatment, which is detrimental to healthy growth and development into adulthood [5]. Large barriers to youth mental health care occur at the organizational and community levels where differing priorities across child-serving agencies may contribute to lower rates of youth access to services [6].

The system of care (SOC) approach was developed in the 1980s as a strategy to address siloed child-serving agencies through an integrated and principle-driven approach to tiered services for youth with social, emotional, and behavioral difficulties [7]. The SOC core values, informed by the Child and Adolescent Service System Program principles [8], are that services should be: (a) family and youth driven, (b) community-based, and (c) culturally and linguistically competent. These values are operationalized through guiding principles such as interagency collaboration, care coordination, and partnerships with families and youth [7]. The SOC approach applies principles to help guide coordinated efforts to support youth whose services intersect multiple child-service agencies (e.g., mental health, judiciary, education, child welfare). Several cross-site studies have evaluated youth SOC efforts over time with differential operational definitions of SOC values and principles [7–11]. Each study indicated the importance of sustainability planning at the outset and aligning infrastructure and service development to meet local system requirements. For example, Brashears and colleagues noted that having interagency involvement in developing and implementing shared administrative processes was a common challenge [9]. Moreover, fiscal crises, leadership turnover, and methodological concerns for assessing long-term sustainment were noted as barriers in the SOC approach. Indeed, the SOC approach requires commitment and financial resources to succeed.

In 1992, the United States federal government signed into public law the establishment of the Substance Abuse and Mental Health Services Administration (SAMHSA; cf. Congressional public law 102–321) given the disconnect between youth and families’ need for services, the SOC approach, and the variable federal financial priorities. The SAMHSA goal was to support substance abuse and mental health prevention and intervention in the United States through the establishment of a federal funding authority operated under the Department of Health and Human Services. Within SAMHSA, there are three major centers that currently fund prevention and intervention services. The Center for Mental Health Services supports the development of services for adults with serious mental illnesses and youth with serious emotional disturbances through the administration and oversight of SOC expansion awards, cooperative agreements, and mental health services block grant programs (i.e., a discretionary fund to help prevent and treat mental health disorders). The Center for Substance Abuse Prevention develops comprehensive prevention systems through national leadership in policy and programs through promoting effective prevention practices and applying prevention knowledge. Their goals are to build supportive workplaces, schools, and communities, drug-free and crime-free neighborhoods, and positive connections with friends and family. Similarly, the Center for Substance Abuse Treatment seeks to improve and expand existing substance abuse treatment and recovery services. This center administers the Substance Abuse Prevention and Treatment Block Grant Program and supports the free treatment referral service to link clients to community-based substance use disorder treatment. The SAMHSA operates an over ten billion a year budget with $225 million dedicated to children’s mental health and SOC initiatives in 2024 [12].

The Hawaiʻi State Department of Health Child and Adolescent Mental Health Division (CAMHD) is the state’s Medicaid behavioral health carveout and the primary agency responsible for developing and administering clinical services for approximately 2,000 youth each year. The CAMHD provides care coordination and clinical oversight at seven regional Family Guidance Centers statewide and delivers in-home (e.g., intensive in-home, Multisystemic therapy) and out-of-home (e.g., transitional family home, community-based residential, hospital-based residential) services through 17 community-based contracted agencies. A centralized state office oversees all administrative, clinical, and performance functions including annual reporting of youth served and clinical outcomes (see https://health.hawaii.gov/camhd/annual-reports/). The CAMHD has a longstanding history of SAMHSA SOC expansion awards beginning in 1994 and continuing to the present in an almost unbroken succession [13]. These developments began shortly after a class-action lawsuit was brought against the state (Felix v. Waihee, 1993), when Hawaiʻi was ranked among the lowest in the nation for youth mental health services [14]. The settlement, referred to as the Felix Consent Decree, resulted in federal oversight that lasted from 1994 to 2004 [15, 16]. The federal decree mandated and oversaw the development of a statewide SOC, and in many ways complemented the goals of SAMHSA SOC expansion awards that overlapped with federal oversight and continued for two more decades. The various SOC awards operationalized SOC principles and ranged from filling in gaps within the service continuum to enhancing existing services through trauma-informed care, wraparound care coordination, and improved knowledge management systems.

The purpose of this study is to examine the Hawaiʻi State CAMHD system’s SAMHSA SOC award history to identify common themes, lessons learned, and recommendations for future funding. The first goal was to understand the development and evolution of SOC values and principles (e.g., youth and family voice) within and across each grant. The second goal was to describe and reflect on common themes and lessons learned through the 30 years and seven CAMHD SOC expansion awards. This is the first study to date that examines themes across previous SAMHSA SOC awards from one state’s perspective. There were no a prior hypotheses given the exploratory nature of this study. The intention was to contribute to research and improved practices around effective SOC grant implementation at the federal and state system levels.

## Methods

This study used a two-phase qualitative approach with (a) content analysis on seven final project reports and (b) key informant interviews with 11 system leaders. Initially, for the final project reports, a matrix template was utilized to summarize data by domains consistent with SAMHSA’s Center for Mental Health Services Infrastructure Development, Prevention, and Mental Health Promotion indicators (e.g., Policy Development, Workforce Development) which would have allowed comparisons across multiple projects and domains. However, after multiple trials to code past project reports into the indicators, the two lead investigators (Okamura, Jackson) opted to use a grounded approach to identifying themes and lessons learned based on SOC values and principles. Initial results from project reports guided the information collected in interviews, which iteratively guided subsequent interviews until saturation and consensus was reached on the final themes.

For the interviews, a purposive sampling strategy was utilized to obtain feedback from system leaders who have had extensive experience within individual grant projects and/or across multiple SOC award projects within the state [17]. Interview participants included four previous grant project directors and seven system leaders whose roles included regional center chiefs (one who, at the time of data collection, was acting as the statewide chief administrator), clinical supervisors, training specialists, and a performance manager. All interviews were recorded and transcribed, except for one participant who declined to be recorded but whose responses were paraphrased in notes. The lead investigators conducted all interviews. A semi-structured interview was developed and used (see Supplemental File), which evolved during the study to further probe more specific themes that were emerging. Initial interview questions asked participants about what they remembered, lessons learned, and what recommendations they had based on the project. Additional probes were used to obtain their perspectives on areas including the project’s impact on the state’s mental health division and larger system of care, its impact on the specific project’s focus areas, and its impact on the division’s relationship with SAMHSA. In addition, participants were asked about their overall reflections on the SOC awards, thoughts on how they have impacted the system over multiple years, and how they could be best utilized in the future.

All data from project reports and interview transcripts were independently coded by the two lead investigators, who each reviewed every report and transcript. Data were analyzed using rapid qualitative analysis techniques [18]. Rapid qualitative analysis is well-suited for projects that aim to be completed in one year or less that do not rely on traditional transcription coding [19]. For this project, main points from interviews were summarized to provide a quick and accessible “sketch” of the data as data were organized and collected. These sketches were organized into a matrix to allow for quick identification of similarities, differences, and trends in responses [20]. ​Therefore, reliability calculations such as kappa or intraclass correlations were not appropriate for this method. This study was deemed exempt and non-human subjects research by the Hawaiʻi State Department of Health Institutional Review Board.

## Results

### System of care principle development and application

The Hawaiʻi State CAMHD has operated seven SAMHSA SOC awards from 1994 to present day (2024) as detailed in Table 1. Several project directors served multiple SOC awards which provided continuity. Specifically, Kealahou, Kaeru, and Data to Wisdom projects had the same project director, which helped to infuse trauma-informed care and bridge previous work in youth and parent partner services. There was variation in the project foci with some projects focused on developing SOC infrastructure (e.g., care coordination model) and others also focused on developing services (e.g., adaptive behavioral intervention) within the service array. The ʻOhana Project and Hoʻomohala both set foundations for the CAMHD SOC by establishing care coordination, contracted provider agencies, and building the service array. Kealahou, Laulima, and Kaeru projects continued to build the CAMHD SOC while focusing on targeted populations and specialty services. The Cultures of Engagement in Residential Care focused primarily on residential treatment settings and eliminating the use of seclusion and restraint. The Data to Wisdom grant focused on SOC development to infuse data driven decision making, knowledge management, and trauma-informed systems. Project geographic locations also changed over time from specific areas (e.g., urban Honolulu) to the broader overall statewide system.

**Table 1:** Hawaiʻi state child and adolescent mental health division SAMHSA awards

Project	Years Project Director Location	Broad Goal	Activities
ʻOhana Project *(Felix consent decree began in 1995)*	1994–2000 Kate Pahinui Waiʻanae Coast, Oʻahu	Promote systems change through the development and demonstration of the SOC approach	• Established SOC • Care coordination model (contracted provider for direct services) and SOC principles • Clarifying roles within child-serving system with Department of Education, Family Court, Office of Youth Services, Hawaiʻi Youth Correctional Facility, and Child Welfare Services • Family voice in governing councils (paid stipends) • Explored Medicaid waiver (managed care landscape)
Cultures of Engagement in Residential Care (4 SM056497)	2004–2008 Lesley Slavin Hawaiʻi residential treatment facilities	Reduce the use of restraint and seclusion for youth within Hawaiʻi’s SOC to the lowest possible level, through a comprehensive set of awareness, training and technical assistance activities supporting service providing agencies and personnel	• Created network of provider agencies through learning collaborative • Created collaboration with family guidance center teams for cross-system learning • Included policy related seclusion and restraint in interagency performance standards and practice guidelines (2006) • Used trauma-informed care model • Included youth and parent involvement
Hoʻomohala (5 SM057063)	2005–2012 David Leake Urban Honolulu, Oʻahu	Culturally and linguistically assess, adapt, develop, evaluate, and sustain a seamless SOC through the integration of a comprehensive array of age-appropriate services and supports and targeted policy and system activities for youth aged 15 to 21 with severe emotional and behavioral disturbance	• Established partnerships with state-level agencies and legislators (e.g., senators, chaired family court judge) • Began using digital media (e.g., television public service announcements, branding and marketing) • Created parent and peer partner support services • Developed categories of engagement based on customer service model to encourage youth to engage in services
Kealahou (U79 SM059024)	2009–2016 Tia Roberts Oʻahu	Build trauma-informed, gender-specific, culturally resonant programming for adolescent (ages 11–18) girls, or those who identify as girls, who have experienced significant trauma and high rates of externalizing and juvenile justice concerns	• Introduced trauma-informed care, training (e.g., learning collaborative), and services (e.g., Seeking Safety) • Focused on non-coercive treatment at youth and family level and interagency education, advocacy, and collaboration • Coordinated state and internal policy development • Created funding strategy through non-profit and through legislative action • Included heavy interagency collaboration within child-serving agencies and community organizations • Leveraged organizational chart to sustain project staff • Created paid positions for youth and parent partners
Laulima (U79 SM061226)	2012–2017 Pratima Musburger Hawaiʻi statewide	Provide accessible, effective and sustainable treatment options and supports for children and youth with co-occurring mental health needs and intellectual/developmental disabilities in their communities	• Collaborative efforts to strengthen partnerships across Hawaiʻi’s child-serving system and to provide training opportunities and technical assistance to families and professionals caring for, and working with children and youth with co-occurring concerns across Hawaiʻi • Established multi agency consent form, formal memorandum of understandings, and independent facilitator contract • Coordinated system- and youth-level dual-diagnosis (e.g., developmental disability) • Used train-the-trainer paradigm to create and spread new Adaptive Behavioral Intervention service • Expanded statutory definition of developmental disabilities
Kaeru (H79 SM063417)	2017–2020 Llasmin Chaine Tia Roberts Hartsock Hawaiʻi statewide (East Hawaiʻi and Honolulu pilot sites)	Return youth who are currently placed in out-of-state residential treatment facilities back to their home communities in Hawaiʻi and prevent impending out-of-state placements	• Explored peer and parent partner certification to embed in service array • Attempted to implement high-fidelity wraparound • Established empirically and data-driven referral method for wraparound • Implemented crisis-text line
Data To Wisdom (H79 SM082961)	2020–2024 Tia Roberts Hartsock Charlene Takeno Hawaiʻi statewide (Kaua’i and Windward Oʻahu pilot sites)	To build a knowledge management approach that infuses various data from youth, provider, and systems to inform decision-making	• Established statewide trauma-informed care taskforce • Provided training in youth clinical decision-making tools for individual youth, therapists, supervisors, and the system • Strengthened interagency collaboration through leadership and fiscal analysis • Created formal training and certification for youth peer partners

Note. SOC = System of Care

### System of care award themes

Content validation and interview coding revealed two content and two general process themes across the seven projects. Content themes were defined as areas that required long-term and consistent efforts across multiple projects and grants to develop. Content themes included (a) interagency collaboration and (b) youth and family voice. Process themes were defined as essential approaches to system improvement work. The general process themes reflected various aspects of (c) connection and (d) continuity, with more specific sub-themes within those.

#### Interagency collaboration

**Fig. 1:**
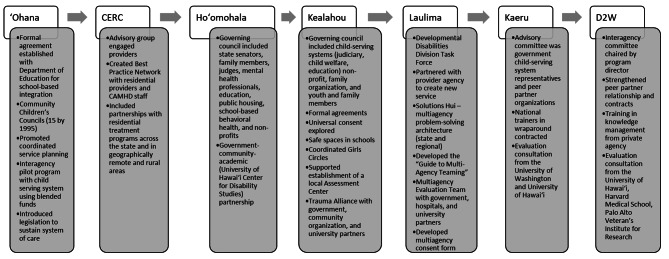
Hawaiʻi state CAMHD interagency collaboration development CAMHD = Hawaiʻi State Child and Adolescent Mental Health Division; CERC = Cultures of Engagement in Residential Care; D2W = Data to Wisdom

The first topic theme reflected the need for continual building of interagency collaboration across every project (see Fig. 1). From the first CAMHD SAMHSA award, the ʻOhana project, CAMHD coordinated interagency agreements with other child serving systems such as the Department of Education and Child Welfare Services. These child-serving system partners served as governing and advisory groups for the SOC awards, alongside consistent integration with other direct service provider agencies and academic partnerships to support the SOC. During Kealahou and Laulima, there was an effort to formalize the interagency collaboration through the execution of memoranda of agreements between agencies and targeted strategies to improve system collaboration, such as the multi-agency consent form. The formation of the Hawaiʻi Interagency State Youth Network of Care through revised statute furthered the commitment to interagency collaboration, which CAMHD and project directors have co-chaired. The development of interagency collaboration followed an advisory (e.g., members from other child-serving agencies contributing feedback to project goals and implementation), integration (e.g., formal advisory council and committee established), and leadership (e.g., chairing advisory council, and leading task forces and special projects) pathway for CAMHD. This theme is consistent with SOC values and principles and align with the priorities across funding announcements to build and enhance SOCs. Perceptions of key informants also reinforced the idea that interagency collaboration was a critical aspect of SOC development; however, successful collaboration is challenging to achieve (see Table 2).

**Table 2:** System of care award themes and quotes

Theme	Quotes
Interagency collaboration	*“That was a big thing, I think, was the impact of utilizing a governing body to come together and collaborate, build relationships with…”*
	*“…if it’s not seen as a priority or if it’s like a limited engagement, like, this is only gonna happen for a year, people are you know, ‘Do I need to make this a priority?’ …there could be a lot of process involved when they have full plates and many other things to do”*
Youth and Family Voice	*“For example, in meetings, there were …things that were being said that were offensive to the youth peers. And so there was a lot of work to prepare and debrief the youth peers after they were in meetings…the secondary traumatic stress and the triggering event at the peers was intense. And so there was a lot of work with that that had to be addressed and done.”*
Connection	*“I think what I want to see is more community building, more family strengthening, more culturally steeped type programs that that build resiliency not just within our families, but within the communities where these things reside. Because when we have healthier communities and healthier families, we have healthier kids.”*
	*“I think the training mechanisms of learning clubs are really important because that creates community. And I think that’s something that is under-recognized how important those collaboratives are, just not one-off, but, like, these long-term, ongoing structures.”*
	*“We had this high-powered governing council, so-called, with the judges chair and a couple of representatives to the senator…representatives from each of the organizations, including [CAMHD], and I think that did help. I think having all those people together, it was sort of a push to formalize, I think, expand, and so I think that was one accomplishment we could say.”*
Continuity	*“…the development of Hawai*ʻ*i Interagency State Youth Network of Care (HISYNC) was a success and I think continues to be a success.”* *“I think the relationships that have been built from there, where people are from those groups, are still working together heavily with me.”*
	*“But, that for me is always the thing. It’s like, how can [we] do better at making these projects sustainable? And not just, you know, these ‘one-off.’ While we have the dollars, we do this, and we have staff, and then, it’s like, once that ends, it’s just ‘poof,’ like, everything’s gone.”*
	*“…it’s hard to see any sort of transformational or long-term change when you have funding for four years, maybe five.”*
	*“…the onus for some of that is on the funder, and if they can do or fund projects that are much longer term. I think a four or five year project is hard.”*
	*“Coordinated service planning isn’t even, like, really a thing anymore…And the medical model part of our system seems to have sort of really changed how we do care coordination, or diminished care coordination in some way.”*
	*“I think that’s the biggest challenge is just the staffing, unless the grant is, from the start, not built around hiring people just for grant work. Or if that is part of it, that you have some sort of transition and matriculation of the duties and responsibilities getting absorbed slowly over time.”*

Note. CAMHD = Child and Adolescent Mental Health Division

#### Youth and family voice

**Fig. 2:**
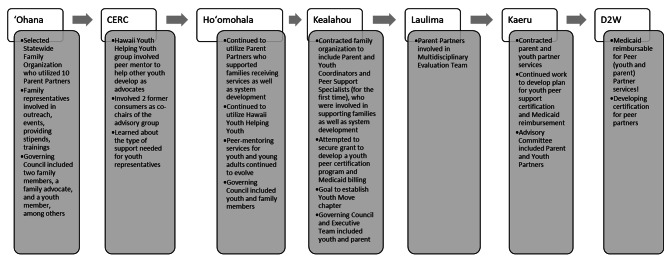
Hawaiʻi state CAMHD youth and family voice development CAMHD = Hawaiʻi State Child and Adolescent Mental Health Division; CERC = Cultures of Engagement in Residential Care; D2W = Data to Wisdom

The second topic theme was youth and family voice, which represents the long road to fully integrating family voice from the system to the client treatment level (see Fig. 2). Parent and youth integration into governing councils and advisory boards to help guide grant activities began from the first award, the ʻOhana project. Eventually, parent and youth peer partner services became integrated into the treatment team level. There were several community-based organizations, like Hawaiʻi Youth Helping Youth, that supported youth and family voice through identifying and training advocates. These advisory activities continued, with more applied support to individual families and youth occurring in Project Kealahou. During this project, the priority to develop a sustainable infrastructure for youth and parent peer partners supporting individual families began. Medicaid reimbursement was pursued for the first time for youth and parent peer partner services, which continued in negotiations to amend the state plan for approximately 12 years. This reimbursement effort continued into the current SOC award, the Data to Wisdom project, with a focus on developing youth peer partner certification as a step toward successful Medicaid reimbursement. Similar to interagency collaboration, the youth and family voice theme progressed from an advisory role (e.g., having youth and families advise grant activities and goals) to informing service (e.g., hiring youth and parent advocates) to pursuing a standalone service (e.g., full integration of youth and parent peer services).

Informant interviews also shed light on the nuances of increasing family voice. New challenges and opportunities emerged alongside greater incorporation of and respect for youth and parent perspectives. One such challenge with youth and family voice is in building trust across different levels within a treatment team and system of care. Language remains a key moderator of trust building (see Table 2). Indeed, the SOC value of youth and family driven and principle of partnership with youth and families were applied differently as youth and parent voice became stronger within the treatment team with the support of peer partners.

#### Connection

Complementing the content themes were process themes related to *how* systems work should be accomplished to be successful, based on the experiences and recommendations of key informants. The first general process theme was encapsulated in the concept of “connection,” as it relates to (1) how services should connect youth to their family, community, and culture, (2) how workforce members should be connected to each other, and (3) how child-serving agencies should be connected to each other. Fostering these connections often goes beyond day-to-day roles and responsibilities and requires additional focused and sustained efforts.

Regarding the connection of youth to their family, community, and culture, one staff member noted a need for community-based interventions (see Table 2). Additionally, connection through communication and relationship building among workforce members and creating the structures to maintain relationships was described as important. One informant noted the importance of learning collaboratives in the project which created a shared place to connect and learn.

Finally, similar to the content theme of interagency collaboration being a continual endeavor, informants relayed many thoughts about how the system could connect agencies together to be more successful in the goal of system improvement. One leadership member noted the need for venues where legislators and other leaders from organizations to come together regularly to discuss issues (see Table 2). The connection not only built trust and clarified roles, but created shared responsibility within the SOC so that not one organization or body was making decisions independently of another.

#### Continuity

The second general process theme was summarized in the concept of “continuity.” This theme emerged from comments about the importance of efforts such as (1) ensuring the continuity (sustaining) of interagency collaboration, (2) ensuring the continuity of new initiatives, (3) ensuring greater continuity (increased length) of award time periods, (4) ensuring continuity in the CAMHD model of care, (5) ensuring continuity in trauma-informed care, and (6) ensuring continuity in staffing. Overall, it was conveyed that better care for youth requires continuity throughout the system, from interagency collaboration to individual treatment.

First, ensuring continuity of interagency collaboration refers to the maintenance of the formal structures and relationships beyond a single project. For example, the establishment of Hawaiʻi Interagency State Youth Network of Care secured a platform for tackling issues that crossed agencies and could function independently from the restraints of single award periods (see Table 2).

More broadly, informants expressed difficulty in achieving sustainability and the need to ensure the continuity of new initiatives instead of them being a “one-off” or pilot projects. Some informants noted that typical award periods are not long enough to develop and sustain successful initiatives. As seen with the youth and family voice topic domain, it does take longer than a single grant to see any sort of transformational or long-term change.

The CAMHD model of care also emerged as a consistent topic throughout the final reports and interviews. The model of care was perceived as a pendulum swinging from a more intensive care coordination model, aligned with system of care values and care coordination principles, to a more “medical model” and managed care. As one person stated “we need to figure out what is our model…” and another informant noted some history related to care coordination to a medical model (see Table 2).

Trauma-informed care was a consistent thread in all SOC awards, and the importance of continuity emerged in interviews. Continuity was critical both at the system level, where consistent efforts needed to be made over multiple grant periods to build a more trauma-informed system, as well as the client level, where addressing a youth’s trauma requires time, patience, and consistency of support from the treatment team. As one person noted:

*“it takes time to do trauma-informed care”* and *“you can only move as fast as the individual is able to move.”*

Finally, a consistent challenge was in ensuring continuity in staffing. With limited award periods, staff begin to find other opportunities when funding nears the end and positions have not become permanent. Moreover, the start of new awards is typically delayed because of the challenges in establishing new positions and hiring new staff. A leadership informant noted that transitioning grant staff to new grants or from existing grants can cause disruptions to the system and staff morale.

## Discussion

The current study was a review on 30 years and seven awards given to the Hawaiʻi State Child and Adolescent Mental Health Division by the Substance Abuse and Mental Health Services Administration to expand the system of care. Two major topic themes of interagency collaboration and youth and family voice were identified that aligned with SOC values and principles. Two process themes of connection and continuity weaved throughout other SOC principles such as trauma-informed care. The Hawaiʻi State CAMHD continues to be a leader in SOC expansion despite ongoing administrative and fiscal challenges that common with other SOC expansion efforts [9, 10]. Their dedication to SOC values and principles is evident in the investment of resources to start and close multiple awards, build interagency collaboration, and innovate within and across the child-serving system and its agencies.

Building interagency collaboration is one of the most difficult aspects of system improvement [9]. The CAMHD has needed to constantly invest resources (e.g., funding, personnel, legislation) to meet its goals. Lessons learned from interagency collaboration range from developments in coordinated interagency agreements with other child serving agencies (e.g., Department of Education, Child Welfare Services) which provided inbuilt advisory groups for SOC expansion, to consistent integration with other direct service provider agencies and academic partnerships to support that expansion, and finally through to formalization and strengthening of interagency collaboration through formal agreements and targeted strategies like the universal and multi-agency consent form. Networking within and between child-serving agencies was noted as an important aspect in building interagency collaboration. However, turnover can impact continuity and momentum. Legislation and policies have the potential to sustain collaboration and must be implemented with intention and proper funding to ensure high quality facilitation informed by equitable methods [21].

Partnering with youth and families has been a consistent theme in successful efforts to expand systems of care in other states, and the CAMHD has sought to continue developing this area through multiple grants despite ongoing challenges [9, 10]. Lessons learned from youth and family voice range from the integration of parent and youth into governing councils and advisory boards, identification and training of advocates, and applied support to individual families and youth including the long and continuing work toward Medicaid reimbursement. It is interesting that the progression from youth and family voice informing service to a standalone service is representative of almost two decades of systems work. Systems change is truly a long-game and there have been many efforts to support these changes, including federal legislation and funding priorities (e.g., SAMHSA Office of Behavioral Equity and new funding priorities around marginalized communities). Moreover, updated SAMHSA funding announcements have explicitly called for language around culturally and linguistically appropriate, evidence-informed, recovery-oriented, trauma-informed care that highlights the commitment around SOC values and principles.

From these lessons, several areas for future attention emerged. These included considerations of the state and federal policies that often seem at odds with each other. As one informant noted: “we need to look at how the contracts and procurement is done.” This is particularly pertinent to state procurement laws which make it difficult to initially collaborate with and contract providers without a suitable means of paying them for their time, further complicating and delaying the work. A key leader noted:

*I think the state system could really benefit from looking at how to support grants better and how to handle rules maybe differently, and procurement differently, and just be, provide more support…I think the state needs a grant office like a, you know, a university would have and they need to help us.*

Moreover, establishing new funding accounts, job descriptions, and personnel management policies intersects divisional, departmental, state, and federal bureaucracies that often contribute to lengthy stalls in completing work and spending funds. For example, for the current SOC award, the project director was hired approximately six months after the notice of award, because the position needed to be established and associated with a new award and account code, despite the person already being in the previous SOC award project director position. Landscaping current federal and state policies on spending, procurement, and community collaboration may help to identify better pathways and strategies to executing federal grants within state infrastructure.

Furthermore, mental and behavioral health payment structures require ongoing attention. Stroul and Manteuffel noted that while award sites reported using a range of financing strategies, increasing Medicaid reimbursement was the most frequent strategy [11]. However, most strategies were not seen as very effective, and the highest effectiveness ratings were for increasing Medicaid funding, increasing state mental health funding, obtaining and coordinating funds with other systems, and redeploying funds to lower cost service alternatives [11]. Certification and credentialing processes that are needed for reimbursement are often time-intensive to develop and requirements may not align with health equity and lived experience. For example, in Project Hoʻomohala, a bachelor’s degree was required to hire a peer specialist. However, this requirement excluded many transitioned-age youth with lived experience who were more closely related in age which may have brokered trust and rapport more quickly. Initiatives that compare funding and certification rates and examine empirically the extent to which financing strategies improve service reach are necessary evaluation activities that should be included in SOC awards [22, 23].

Programs for targeted populations and complex cases, which allow for flexible scheduling and funding, are also needed. Co-occurring mental health, disabilities, and substance use programs provide holistic care for youth and families. Special populations like racial, ethnic, sexual orientation, and gender/sex minorities that require adapted interventions should be a federal and state funding priority. As one interviewee noted:

*Girls matter. Treatment for girls needs to be individualized more so than just, I don’t know, some of the EBS [evidence-based services] stuff you know, and I’m not knocking the EBS stuff, that is important. We need more research about girls. And that is a recommendation…The basic need is huge, so I think the lessons learned, we really do need more flexible funding to be able to support girls in their treatment, girls in their homes.*

Improving integration into existing structures like home-based care, primary care, and school-based services, as well as integration of informal supports (e.g., youth peer support), requires continued effort to evolve with the changing managed care landscape. Payment and reimbursement strategies to incentivize practice use and improved clinical outcomes should also be considered.

Several recommendations emerged from the current study for operating future SAMHSA SOC awards in CAMHD and other state systems. First, there was enthusiasm for the focus of SOC awards to include more goals around infrastructure development and sustainment and to avoid “stand-alone” services. For example, one informant noted that “*It’s kind of a problem if you have a stand-alone service with its own team and it’s going to go away when the grant money is gone.*” Indeed, sustainability planning should begin prior to an infrastructure grant application being written to ensure there is a clear sustainable financial plan or objectives to continue pursuing funding for specific initiatives. Integrating procurement and administrative activities as specific and targeted award objectives, while unconventional, will emphasize the disconnect between federal and state procedures and spending priorities. Both state and federal legislators should be aware of funding mechanisms that have the potential to operate well in state government and to champion legislation that would create less bureaucracy in favor of the community. For example, including procurement clauses within federal funding announcements that allow for the federal government to supersede state laws may aid in timely execution of contracts using federal funds. Moreover, creating grants management, contracting, and fiscal positions that sit within procurement and administrative offices at the highest department level will be crucial to more timely execution of grant activities. Second, reliance on within-system historical knowledge is fraught with error. Future SOC awards should include evaluation objectives, like this project, to memorialize previous accomplishments, reflect on shared understanding and inconsistencies, and to archive important SOC activities in legacy documents. The third recommendation is related to communication within and between SOC awards by maintaining staff from one project to another. It is helpful to have ongoing role and responsibility clarification meetings internally and with child-serving system partners to avoid confusion and miscommunication. Learning collaboratives and protected time for project directors to share lessons learned and recommendations would aid in knowledge consolidation between projects. It would also be beneficial to allow for multi-year overlap of federal SOC awards to create continuity and retain employees. As one informant noted:

*“And we recruited and hired a lot of really great people, and I think that the challenge becomes, as the grant starts to come to a close, or is nearing its end, that you recognize that people may leave because the positions are time limited. So, to the extent that it’s possible to think about positions for those folks, I feel like that is important.*

Trauma-informed care principles are a necessary component of ensuring continuity of care. Trauma-informed care requires active responses in the form of integrating knowledge related to trauma into policies, procedures, and practices as well as careful attention to avoiding re-traumatization and secondary trauma of those involved in systems [24]. Lessons learned include making changes at the individual and organizational level to ensure that all aspects of care would be both transparent and trauma-informed. As one informant noted:

*For example, in meetings, there were things that were being said in, in care coordination meetings, things that were being said that were offensive to the youth peers. And so there was a lot of work to prepare and debrief the youth peers after they were in meetings. We had peers that had previously been in care and saw their, their, their staff that they had worked with in some of our meetings. I mean, and had really negative experiences with them. And so the debriefing and you know, the secondary traumatic stress and the triggering even at the peers was intense. And so there was a lot of work with that that had to be addressed and done.*

Guiding principles of trauma-informed care include creating a safety net instilled with trustworthiness and transparency, among others, to build confidence toward motivation for continued engagement [25]. Moreover, and consistent with interagency collaboration, a trauma-informed child-serving system should create a shared lexicon that speaks within and between agencies to improve navigation for youth and families. One informant noted:

*And thinking about the needs of children as complex and they may have needs that span the way government agencies are organized. And so, recognizing that it is on the onus of government or organizations to be set up to better serve families rather than the onus on families to try to navigate really burdensome infrastructure to get the services that they need.*

### Limitations

The current study is not without limitations. First, the study relied on retrospective accounts of past project final reports and informant interviews. Both sources of information included objective and subjective accounts of previous SAMHSA SOC awards that represent a limited perspective. Moreover, key informants were identified via purposive sampling which may affect generalizability to other systems. Future research may wish to focus on convergence with multiple sources of objective data including financial reports, progress indicators, and any other technical assistance data available. Second, the information sources rely heavily on leadership and a small subgroup of CAMHD staff perspectives. It is unclear the extent to which some of these themes and lessons learned are uniformly understood throughout the various levels and roles within CAMHD and the child serving system. Additionally, the initial content coding design intended to rely on SAMHSA Infrastructure Development, Prevention, and Mental Health Promotion indicators (e.g., Policy Development, Workforce Development) to aid in generalizability to other systems. However, the indicator definitions were difficult to operationalize. For example, the Workforce Development categories contain five indicators that measure the number of organizations, communities, people, changes, and consumer or family members that are trained in, are credentialed or certified, implemented, and/or delivered mental health services. However, these metrics were almost never reported on within final reports and it was unclear how meaningful these metrics were to the system and aligned with SOC values and principles. While this study ultimately chose to use a grounded approach, future studies may wish to carefully think through key indicators to compare within and across systems over time. Despite these limitations, examining SAMHSA SOC awards within one system has the potential to inform how state and federal governments operate funds to support mental health innovation. Additional methods like landscape analysis and policy development could help to address the financial and administrative bureaucracy of operating federal funds in a state government association.

## Conclusions

Federal funding is critical to addressing the youth mental health crisis [4]. The current study examined system of care expansion trends that represented multimillion-dollar investments and decades of work around interagency collaboration and youth and family voice, as well as attempts to build connection and continuity. It is hoped that the lessons learned will aid other systems and future work in being more evidence-informed. Similar delays in award progress and spending stemming from incongruencies between state and federal policies are consistent with previous SOC research and anecdotal reports from others involved in SAMHSA and SOC efforts. Targeted state alignment with federal policies and longer funding mechanisms may aid in ameliorating administrative burden on systems. That said, SAMHSA SOC expansion awards have the potential to fund innovative work that create legacy cultures around SOC values and principles.

### Electronic supplementary material

Below is the link to the electronic supplementary material.

Supplementary Material 1

## Data Availability

The dataset used and/or analyzed during the current study are available from the corresponding author on reasonable request.

## Abbreviations

CAMHD: Child and Adolescent Mental Health Division
CERC: Cultures of Engagement in Residential Care
D2W: Data to Wisdom
HISYNC: Hawaiʻi Interagency State Youth Network of Care
SAMHSA: Substance Abuse and Mental Health Services Administration
SOC: System of Care
