# Supplementary material for: 30 years of youth system of care lessons learned – a qualitative study of Hawaiʻi’s partnership with the Substance Abuse and Mental Health Services Administration
Source: BMC Health Serv Res. 2024 May 23;24:658. doi: 10.1186/s12913-024-11114-9 (PMC11118110; doi:10.1186/s12913-024-11114-9)
Supplement: Supplementary file 1 — Supplementary Material 1 [file 12913_2024_11114_MOESM1_ESM.docx]

CAMHD SAMHSA Lessons Learned Semi-Structured Interview

Name:

Date:

Verbal consent to record:

Thank you for taking the time to meet with me today. As part of the current SAMHSA system of care award, Data to Wisdom, we are interviewing key informants that have historical knowledge of past SAMHSA grants and project activities. Our goal is to understand the cross-cutting themes across SAMHSA awards, examine lessons learned, and hear your recommendations for future CAMHD efforts.

Your participation is voluntary in this project. We will be recording the interview primarily for reference and your individual thoughts will not be shared outside the research and evaluation team. Aggregate themes will be presented in various outlets, including but not limited to, CAMHD internal meetings, CAMHD provider meetings, CAMHD system partner meetings, and local or national research and evaluation conferences.

**Part I. Lessons Learned**

Our evaluation team reviewed past SAMHSA final reports to identify project specific themes and lessons learned. We will review each project and highlight broad goals, themes, and lessons learned. We will then follow up with additional questions regarding each project. When we ask questions about the “system of care” we are referring to not only CAMHD but the additional child-serving agencies that intersect with CAMHD youth. For example, that might include Department of Education, Child Welfare Services, or the Judiciary.

‘Ohana Project

1. What do you remember from this project? *(Probe: What additional thoughts do you have?)*
2. What are some lessons learned?
   1. *(Probe: How did the project impact the system of care?)*
   2. *(Probe: How did the project impact CAMHD?)*
   3. *(Probe: How did the project affect the content and/or focus-area?)*
   4. *(Probe: How did the project affect CAMHD working with SAMHSA?)*
3. Any recommendations based on this project?

Cultures of Engagement in Residential Care (CERC)

1. What do you remember from this project? *(Probe: What additional thoughts do you have?)*
2. What are some lessons learned?
   1. *(Probe: How did the project impact the system of care?)*
   2. *(Probe: How did the project impact CAMHD?)*
   3. *(Probe: How did the project affect the content and/or focus-area?)*
   4. *(Probe: How did the project affect CAMHD working with SAMHSA?)*
3. Any recommendations based on this project?

Ho‘omohala

1. What do you remember from this project? *(Probe: What additional thoughts do you have?)*
2. What are some lessons learned?
   1. *(Probe: How did the project impact the system of care?)*
   2. *(Probe: How did the project impact CAMHD?)*
   3. *(Probe: How did the project affect the content and/or focus-area?)*
   4. *(Probe: How did the project affect CAMHD working with SAMHSA?)*
3. Any recommendations based on this project?

Kealahou

1. What do you remember from this project? *(Probe: What additional thoughts do you have?)*
2. What are some lessons learned?
   1. *(Probe: How did the project impact the system of care?)*
   2. *(Probe: How did the project impact CAMHD?)*
   3. *(Probe: How did the project affect the content and/or focus-area?)*
   4. *(Probe: How did the project affect CAMHD working with SAMHSA?)*
3. Any recommendations based on this project?

Laulima

1. What do you remember from this project? *(Probe: What additional thoughts do you have?)*
2. What are some lessons learned?
   1. *(Probe: How did the project impact the system of care?)*
   2. *(Probe: How did the project impact CAMHD?)*
   3. *(Probe: How did the project affect the content and/or focus-area?)*
   4. *(Probe: How did the project affect CAMHD working with SAMHSA?)*
3. Any recommendations based on this project?

Kaeru

1. What do you remember from this project? *(Probe: What additional thoughts do you have?)*
2. What are some lessons learned?
   1. *(Probe: How did the project impact the system of care?)*
   2. *(Probe: How did the project impact CAMHD?)*
   3. *(Probe: How did the project affect the content and/or focus-area?)*
   4. *(Probe: How did the project affect CAMHD working with SAMHSA?)*
3. Any recommendations based on this project?

Data to Wisdom

1. What do you remember from this project? *(Probe: What additional thoughts do you have?)*
2. What are some lessons learned?
   1. *(Probe: How did the project impact the system of care?)*
   2. *(Probe: How did the project impact CAMHD?)*
   3. *(Probe: How did the project affect the content and/or focus-area?)*
   4. *(Probe: How did the project affect CAMHD working with SAMHSA?)*
3. Any recommendations based on this project?

**Part 2. Overall Reflections**

Given the past SAMHSA and CAMHD projects we’ve just talked about, overall, what are some reflections on CAMHD’s system of care awards?

(*Probe: How should CAMHD best utilize SAMHSA grants in the future?)*

**Part 3. Closing Question**

Is there anything else that we haven’t talked about that you would like to share? *(Probe: Perhaps a candid thought or memory related to your work or experience with the project(s)?)*

**Additional notes if needed:**

We used the SAMHSA Infrastructure Development, Prevention, and Mental Health Promotion (IPP) indicators guide to code domains
